# New neurons in old brains: implications of age in the analysis of neurogenesis in post-mortem tissue

**DOI:** 10.1186/s13041-022-00926-7

**Published:** 2022-05-02

**Authors:** Dylan J. Terstege, Kwaku Addo-Osafo, G. Campbell Teskey, Jonathan R. Epp

**Affiliations:** Department of Cell Biology and Anatomy, Hotchkiss Brain Institute, Cumming School of Medicine, University of Calgary, HMRB 162, Health Sciences Centre, 3330 Hospital Drive NW, AB T2N 4N1 Calgary, Canada

## Abstract

Adult neurogenesis, the proliferation and integration of newly generated neurons, has been observed in the adult mammalian hippocampus of many species. Numerous studies have also found adult neurogenesis in the human hippocampus, but several recent high-profile studies have suggested that this process is considerably reduced in humans, occurring in children but not in adults. In comparison, rodent studies also show age-related decline but a greater degree of proliferation of new neurons in adult animals. These differences may represent biological species differences or could alternatively be explained by methodological differences in tissue handling and fixation. Here, we examine whether differences in the post-mortem interval between death and tissue fixation might impact subsequent detection of adult neurogenesis due to increased tissue degradation. Because there are fewer new neurons present in older subjects to begin with we hypothesized that, subject age might interact significantly with post-mortem interval in the detection of adult neurogenesis. We analyzed neurogenesis in the hippocampus of rats that were either perfusion-fixed or the brains extracted and immersion-fixed at various post-mortem intervals. We observed an interaction between animal age and the time delay between death and tissue fixation. While similar levels of neurogenesis were observed in young rats regardless of fixation, older rats had significantly fewer labeled neurons when fixation was not immediate. Furthermore, the morphological detail of the labeled neurons was significantly reduced in the delayed fixation conditions at all ages. This study highlights critical concerns that must be considered when using post-mortem tissue to quantify adult neurogenesis.

**Supplementary information:**

The online version contains supplementary material available at 10.1186/s13041-022-00926-7.

## Background

Adult neurogenesis occurs in the mammalian brain in at least two main regions, the hippocampal dentate gyrus and the subventricular zone. Among mammalian species, neurogenesis has been most widely studied in rodents. In rodents neurogenesis declines with age but, remains detectable throughout adult life. Adult hippocampal neurogenesis has been observed in most mammalian species[1]. However, in humans, there exists some disagreement whether postnatal neurogenesis occurs in adults or just in children. Adult neurogenesis in humans was first observed in cancer patients administered Bromodeoxyuridine to track tumor growth[2]. Post-mortem analysis indicated continued neurogenesis in adults. Numerous studies have confirmed these findings using brains ranging from 0 to 100 years old, using markers of proliferation and of immature neurons (For review see[3]). However, several recent papers reported the absence of adult generated neurons in humans although they did observe neurogenesis in juveniles[4–7].

An important consideration when analyzing adult neurogenesis in humans relates to harvesting of tissue and the degree to which the tissue may degrade prior to fixation. In most cases, human tissue is collected post-mortem and the time-window between death and tissue collection is highly variable. The longer it takes to initiate fixation the greater the degree of tissue/protein degradation that may occur[8–10]. In addition, penetration of the fixative when an immersion technique is employed, is not instantaneous and depending on tissue size it can take hours for the interior aspects of the tissue to be exposed to fixative. The impact of PMI on neurogenesis detection has been qualitatively assessed in rats across several PMIs at a single age[11]. This effect has also been examined in human brain tissue, albeit without a zero-delay control group[12]. Although PMI and neurogenesis were not significantly correlated this study did not consider the effect of age. Given that protein synthesis and degradation are often regulated by age[13] there is a potential interaction between PMI and subject age in the ability to detect neurogenesis related protein using immunohistochemistry. Rodent studies allow for highly controlled tissue collection using perfusion fixation to obtain relatively rapid, and consistent fixation. This provides a suitable control group to examine the impact of PMI interval on detection of Doublecortin, a common proxy marker of adult neurogenesis.

## Methods

**Fig. 1:**
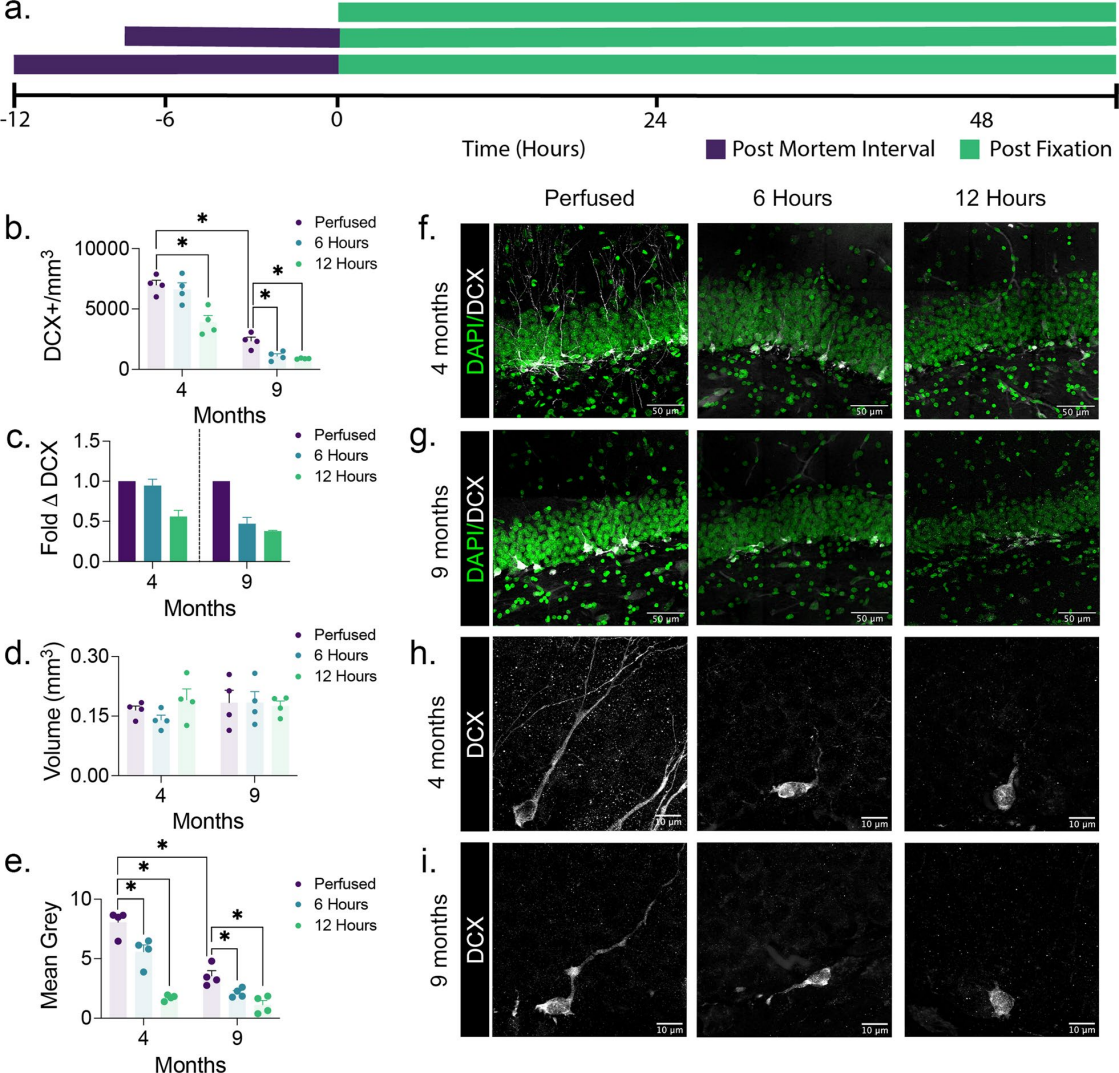
Doublecortin labeling is reduced with increased post-mortem interval. **a** Following post-mortem intervals of 0, 6, or 12 h, brains from 4- and 9-month-old rats (*n* = 4 were fixed for 48 h. The effects of age and post-mortem interval interacted significantly in influencing the **b** density of doublecortin^+^ cells (Two-Factor ANOVA, Age × Post-Mortem Interval Interaction; F_2,18_ = 5.448, *p* = 0.0141), **c** which we have also presented as a fold difference in doublecortin^+^ cell density relative to the mean density of the perfused groups of each age (Two-Factor ANOVA, Age × Post-Mortem Interval Interaction; F_2,18_ = 4.478, p < 0.0264). **d** This change in density of doublecortin^+^ cells occurred without any differences in dentate gyrus volume. **e** Age and post-mortem interval also interacted significantly to influence the optical density of doublecortin labelling in the granule cell layer (Two-Factor ANOVA, Age × Post-Mortem Interval Interaction; F_2,18_ = 12.34, *p* = 0.0004). Representative images of doublecortin^+^ cells in the subgranular zone in 4-month-old (**f**) and 9-month-old (**g**) rats with post-mortem intervals of 0, 6, and 12 h. Representative images of the morphology of doublecortin labelling in 4-month-old (**h**) and 9-month-old (**i**) rats with post-mortem intervals of 0, 6, and 12 h. Data are mean ± SEM. Post-hoc analyses of significant interactions in Two-Factor ANOVA used Newman Keuls. *p < 0.05

We examined brains from 4-month or 9-month-old male adult Sprague Dawley rats that were transcardially perfused to age matched rats that were killed, and their brains collected 6, or 12 h post-mortem. Rats in the perfused group were deeply anesthetized using isoflurane and perfused with 60 ml of PBS followed by 120 ml of 4% formaldehyde. Extracted brains were immersion fixed in 4% formaldehyde for 48 h. For the delay fixation conditions, rats were given a lethal overdose of isoflurane (5% ISO delivered in 2% oxygen in an induction chamber, until death) and the brains were extracted following a delay of either 6 or 12 h, during which time carcasses were kept at room temperature (Fig. 1a). Brains were placed in 4% formaldehyde for 48 h and then transferred to 30% sucrose prior to sectioning on a Cryostat (Leica CM1950) at a thickness of 40 μm. Tissue series (1/12) were stored at -20 °C in antifreeze.

Doublecortin labeling was performed using a 1:250 dilution of primary antibody (Cell Signalling 4601 S Rabbit anti-doublecortin) followed by a 1:500 dilution of donkey anti-rabbit Alexa Fluor 488 secondary antibody (Jackson Immuno Research Laboratories AB_2338072). Quantification was performed on an Olympus BX63 fluorescent microscope at 60x magnification. Labeled cells were counted through the entire dentate gyrus and were included if the cell body could be clearly identified and was located in the granule cell layer or subgranular zone. Doublecortin counts were normalized by the linear distance of the subgranular zone, which was traced in *ImageJ* using DAPI labelling as reference. Slides were coded and quantification was performed blind to treatment.

The optical density of doublecortin labelling in the outer half of the granule cell layer was performed on an Olympus FV3000 confocal microscope using a 10x objective. Three images, each from different tissue sections, were collected at a z-spacing of 3.96 μm. Illumination and z-range were consistent across all images. Background mean grey pixel intensity values of the z-projected doublecortin images were recorded from the outer molecular cell layer using *ImageJ*. Background intensity was subtracted from mean pixel intensity recorded in the upper blade of the dentate gyrus.

## Results

We predicted that post-mortem protein degradation might have an age-dependent influence on the detection of adult generated neurons with a greater influence on detection in older brains. Our results confirm this prediction (Fig. 1b, c). We found a significant age by fixation interaction. Post-hoc analysis of the data indicated the expected reduction in doublecortin between 4 months and 9 months in perfused rats. Importantly, there was a significant impact of the post-mortem interval on our ability to detect doublecortin. At the shortest delay of 6 h there was no significant difference between perfused and non-perfused 4-month-old rats. However, in 9-month-old rats there was a significant decrease in doublecortin + cells counted in non-perfused rats. At 12 h there were significant decreases in doublecortin + cells counted in both 4- and 9-month-old rats.

We also qualitatively examined the appearance of doublecortin-labeled cells in each condition. Perfusion fixation resulted in labeled cells with clear morphology and consistent labeling, while delayed fixation compromised morphological detail. Some cell bodies appear condensed (and subsequently brightly labeled). In others, the normal pattern of cytoplasmic doublecortin labeling was present but often appeared weak and speckled following delayed perfusion, rendering identification of new neurons increasingly difficult. These observations were supported by decreased optical density of doublecortin staining in the outer granule cell layer of the dentate gyrus (Fig. 1e). A significant age by fixation interaction was present in the quantified optical density of this region. This simple analysis does not distinguish between degraded dendritic labeling versus fewer labeled cells but adds support to the overall finding of an interaction between PMI and age in the detection of neurogenesis.

In conclusion, we demonstrate that age related differences in neurogenesis in post-mortem tissue should be interpreted with caution as there is potential for misinterpretation of decreased or absent neurogenesis in older subjects. We speculate that the interaction between age and fixation could be driven by a decrease in doublecortin protein expression in the immature cells of older animals. Protein synthesis is often decreased with age[13]. To our knowledge, it is not known if there is reduced doublecortin expression in new neurons from aged subjects, but other proliferation related genes in doublecortin-expressing cells are known to be reduced[14]. Doublecortin may be detectable with optimized fixation and antigen retrieval, but due to lower protein levels, may fall below detection limits[12, 15]. While our results do not directly show the effect of PMI on histological quantification in humans, we provide reason to cautiously interpret histological reports following PMIs. The extent to which such an effect may be observed with other proteins is unclear but should be considered in future studies. It is possible that doublecortin may degrade more rapidly than other proteins which may be more stable in the detection of neurogenesis in post-mortem tissues. While this is currently unknown, our approach provides a strategy to determine the age-related stability of different proteins prior to post-mortem analysis.

## Supplementary Information

**Additional file 1. Supplementary data** Source data for Figure 1.

## Data Availability

The data that support the findings of this study are available in the manuscript and available from the corresponding author upon reasonable request.
